# Microfluidic-assembled curcumin ionizable lipid nanoparticles enhance aqueous delivery and antibiofilm activity against *Staphylococcus aureus*

**DOI:** 10.1016/j.ijpx.2026.100630

**Published:** 2026-08-03

**Authors:** Xin Jin, Zhentao Xing, Geng Yang, Harrie Weinans, Jaqueline Lourdes Rios, Joost P.G. Sluijter, Raymond M. Schiffelers, Zhiyong Lei, Zijian Ye

**Affiliations:** a Laboratory of CDL Research, University Medical Center Utrecht, Utrecht 3508 GA, the Netherlands; b Department of Urology, University Medical Center Utrecht, Utrecht 3584 CX, the Netherlands; c Laboratory of Experimental Cardiology, Department of Heart & Lungs, University Medical Center Utrecht, Utrecht 3584 CX, the Netherlands; d Department of Orthopaedics, University Medical Center Utrecht, Utrecht 3584 CX, the Netherlands; e UMC Utrecht Regenerative Medicine Center, Circulatory Health Research Center, University Medical Center Utrecht, Transplantation Center Utrecht, Utrecht University, Utrecht 3508 GA, the Netherlands

**Keywords:** Curcumin, Ionizable lipid nanoparticles (LNP), Microfluidic assembly, *Staphylococcus aureus*, Biofilm, Extracellular polymeric substances, Aqueous solubilization

## Abstract

Antimicrobial resistance and biofilm-associated infections demand novel therapeutic strategies. Curcumin is a natural polyphenol with broad-spectrum antimicrobial activity; however, its clinical utility is fundamentally constrained by negligible aqueous solubility and limited biofilm penetration. Here, we used microfluidic assembly to prepare curcumin-loaded ionizable lipid nanoparticles (Cur@LNP) with three clinically established ionizable lipids (SM-102, MC-3, and ALC-0315). The resulting formulations were uniform, with hydrodynamic diameters of approximately 90 nm and polydispersity indices near 0.1. Relative to free curcumin in DMSO, Cur@LNP lowered the minimum inhibitory concentration (MIC) 8-fold and the minimum bactericidal concentration (MBC) 16-fold against planktonic *Staphylococcus aureus*, and markedly reduced viable biofilm-resident cells at matched concentrations. Scanning electron microscopy showed that Cur@LNP treatment remodeled mature biofilms into irregular, debris-rich aggregates with reduced extracellular matrix coverage. Notably, antimicrobial performance was indistinguishable across the three structurally distinct ionizable lipids at every concentration tested. Within this in vitro, single-strain system, the antibiofilm effect is therefore dominated by the cargo (curcumin) rather than by ionizable-lipid chemistry, and it is most likely attributed to enhanced aqueous solubilization of curcumin rather than to engagement of the lipid headgroup with the biofilm matrix. These results position microfluidic-assembled ionizable LNPs as a reproducible, scalable solubilization platform for poorly water-soluble natural antimicrobials.

## Highlights

• Microfluidic ionizable LNPs render curcumin aqueous-deployable against *S. aureus*.
• Cur@LNP lowered MIC 8-fold and MBC 16-fold versus free curcumin.
• Three distinct clinical ionizable lipids gave indistinguishable effects.
• Antibiofilm activity is cargo-dominant, reflecting curcumin deployment.

## Graphical abstract

Graphical abstract

**Figure:**
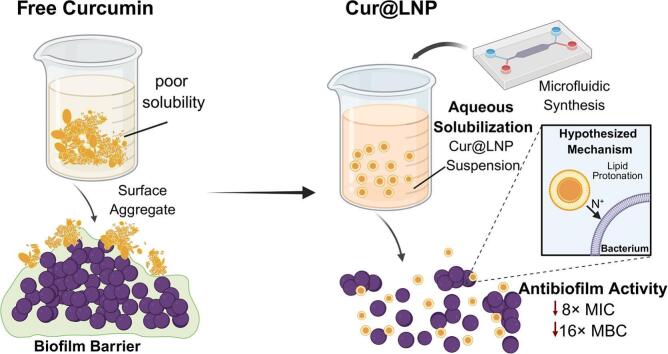

## 1 Introduction

Antimicrobial resistance (AMR) is now widely recognized as a serious global health concern, steadily eroding the effectiveness of conventional antibacterial therapies and adding to the burden of persistent, difficult-to-resolve infections (Ahmed et al., 2024; Marston et al., 2016; Naghavi et al., 2024; O'Neill, 2016). *Staphylococcus aureus* (*S. aureus*) continues to rank among the leading causes of both healthcare-associated and community-acquired infections, especially those linked to indwelling medical devices and implanted biomaterials (Tong et al., 2015; van Loo et al., 2007). In such environments, bacterial persistence is closely tied to biofilm formation: once established, these surface-associated communities are sheltered within a protected niche where the extracellular polymeric substances (EPS) matrix limits antimicrobial penetration, while metabolic heterogeneity gives rise to tolerant or quasi-dormant subpopulations that respond poorly to conventional antibiotics (Anwar and Costerton, 1990; Flemming and Wingender, 2010; Jamal et al., 2018; Koo et al., 2017; Tong et al., 2015).

Curcumin, a natural polyphenol isolated from *Curcuma longa*, has attracted considerable attention because of its antimicrobial, antioxidant, anti-inflammatory, and antibiofilm activities (Adamczak et al., 2020; Cai et al., 2022; Dai et al., 2022; Hettiarachchi et al., 2022; Hussain et al., 2022). Despite this promise, its practical application remains severely restricted by extremely poor aqueous solubility, reported to be below 0.125 mg/L, as well as limited dispersibility in physiological media and low bioavailability at the target site (Datta et al., 2018; Feng et al., 2017; Le et al., 2025). A variety of nanocarrier systems have been investigated to improve curcumin's solubility and antimicrobial performance, including liposomes (Bhatia et al., 2021; Wu et al., 2022), solid lipid nanoparticles (Sandhu et al., 2021), chitosan-based nanoparticles (Ma et al., 2020), mesoporous silica platforms (Barros et al., 2020; Medaglia et al., 2024; Memar et al., 2025), and metallic nanoparticles (Salama et al., 2024). While these platforms can improve curcumin's aqueous solubility and antibiofilm performance, they vary considerably in the reproducibility and scalability of particle production, and most rely on passive solubilization. Beyond curcumin, nanocarrier encapsulation has been widely explored to potentiate antibiotics and other antimicrobials against drug-resistant and biofilm-associated staphylococcal infections (Oliva et al., 2023). Notably, microfluidic-assembled ionizable lipid nanoparticles, the platform that enabled the clinical translation of mRNA vaccines (Wei et al., 2022; Zhang et al., 2021), have not been systematically evaluated in this antibiofilm context. Microfluidic assembly is attractive because it produces particles with narrow, reproducible size distributions (Sato et al., 2016) using clinically established lipids and is amenable to scalable production (Haque et al., 2024); whether the ionizable lipid contributes beyond solubilization is an open question that the present study was designed to begin addressing.

Ionizable lipid nanoparticles (LNPs) are a clinically validated delivery platform, as evidenced by their central role in mRNA vaccines (Estapé Senti et al., 2024; Jeong et al., 2023; Wei et al., 2022; Zhang et al., 2021). These systems utilize pH-responsive lipids that remain neutral at physiological pH but undergo protonation in acidic microenvironments (Eygeris et al., 2022), a property that has prompted the hypothesis that such carriers might also engage the mildly acidic microenvironment reported within bacterial biofilms (Hidalgo et al., 2009; Kiamco et al., 2017). Earlier work has shown that ionizable LNP formulations can alter biofilm morphology and contribute to matrix destabilization in a model system using gold-nanocluster-loaded LNPs (AuNCs@LNP) (Ye et al., 2026). That observation raised the possibility that the carrier itself, and not only its cargo, might contribute to biofilm interactions.

**Scheme 1:**
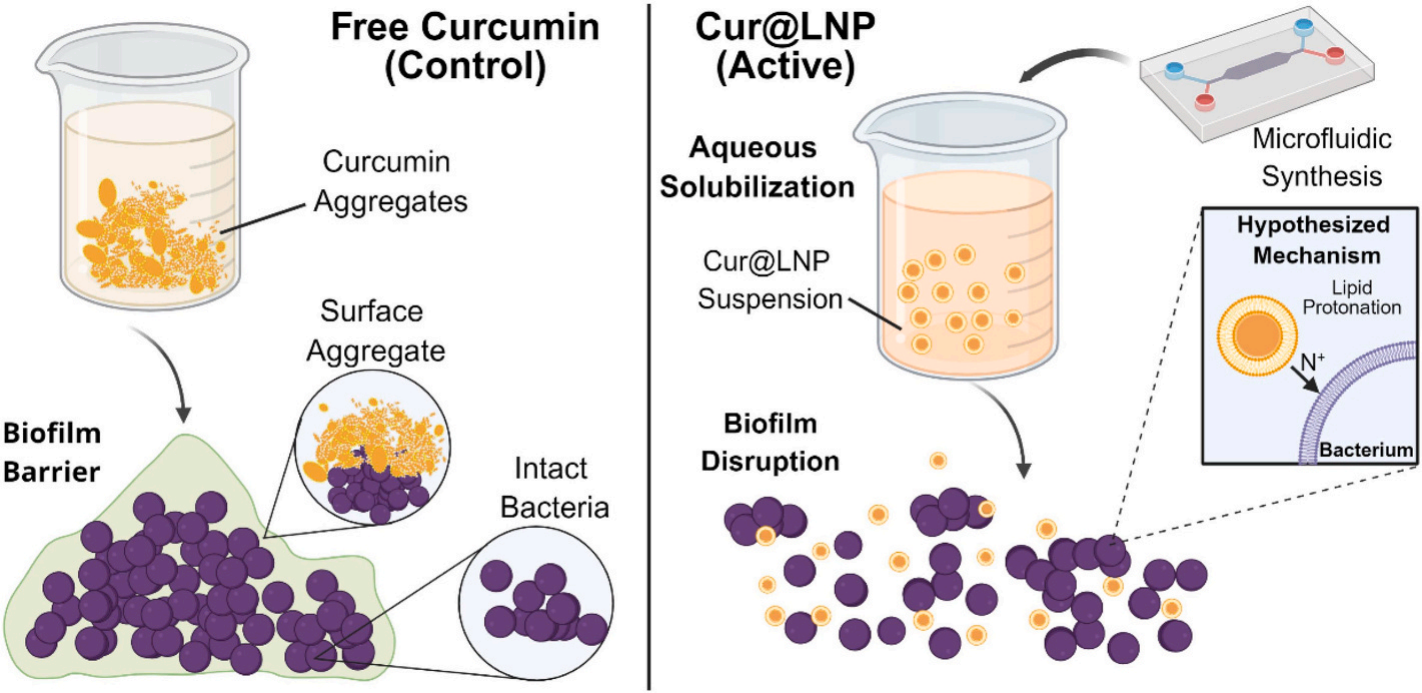
Proposed basis for Cur@LNP-mediated disruption of *Staphylococcus aureus* biofilms. Free curcumin is poorly soluble and leaves biofilms largely intact, whereas microfluidic-assembled Cur@LNP enables aqueous solubilization of curcumin and is associated with biofilm dispersion. The inset shows a hypothesized mechanism, shown for illustration only.

In this work, we used microfluidic assembly to develop curcumin-loaded ionizable lipid nanoparticles (Cur@LNP) and evaluated their efficacy against mature *S. aureus* biofilms (Scheme 1). We tested two hypotheses: first, that microfluidic assembly with ionizable lipids would generate aqueous-deployable curcumin nanoparticles able to lower the MIC and disrupt mature *S. aureus* biofilms relative to free curcumin in DMSO; and second, that the choice of ionizable lipid (SM-102, MC-3, or ALC-0315) would influence the antibiofilm outcome. Within this in vitro, single-strain system, our data consistently support the first hypothesis. The three clinically used lipids gave comparable outcomes, consistent with a cargo-dominant mechanism in which the lipid mainly renders curcumin aqueous-deployable; the proposed pH-responsive engagement of the ionizable headgroups therefore remains a hypothesis rather than an established mechanism.

## 2 Results

### 2.1 Physicochemical characterization of Cur@LNP formulations

**Fig. 1:**
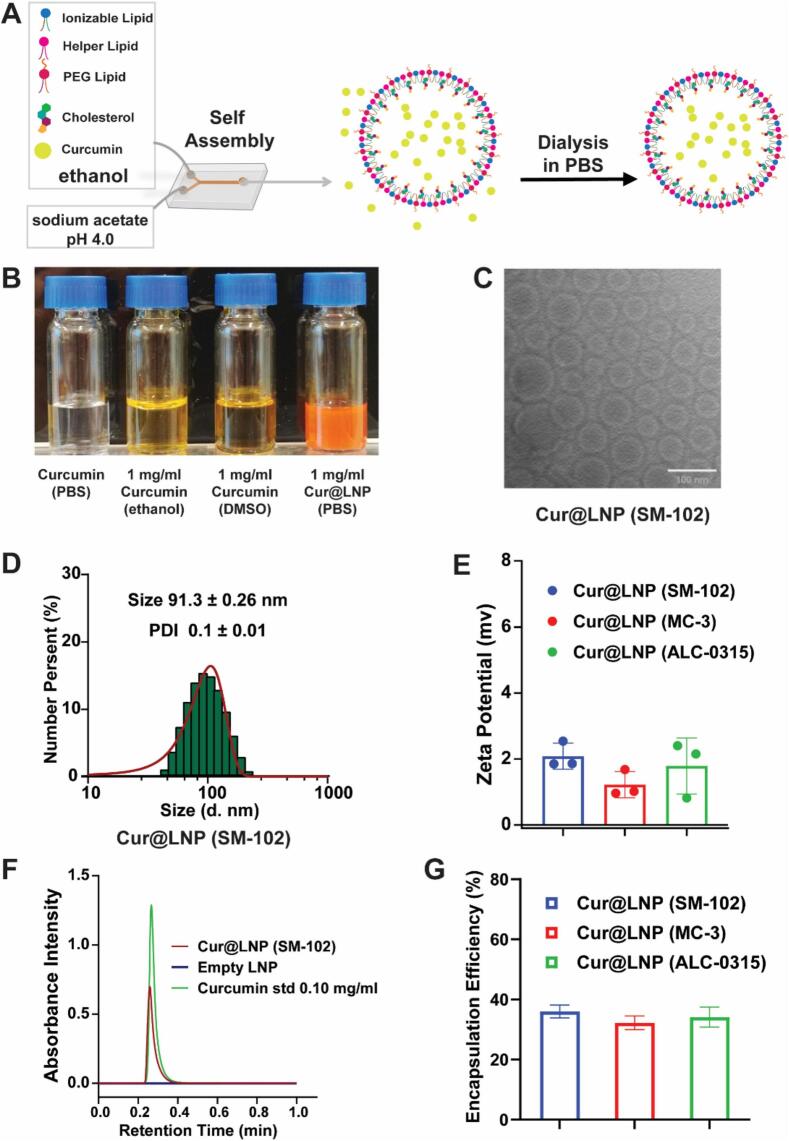
Physicochemical characterization of Cur@LNP formulations. (A) Schematic illustration of the microfluidic assembly process used to prepare Cur@LNP. (B) Macroscopic appearance of free curcumin in PBS, curcumin in ethanol, curcumin in DMSO, and Cur@LNP (1 mg/mL) in PBS after centrifugation. (C) Representative cryo-TEM micrographs of Cur@LNP(SM-102), scale bars: 100 nm. (D) Hydrodynamic diameter distributions measured by dynamic light scattering (DLS). (E) Zeta potential values measured in PBS (mean ± SD, *n* = 3). (F) Representative UPLC chromatograms of Cur@LNP and empty LNP (detection at 425 nm). (G) Encapsulation efficiency of curcumin in the three Cur@LNP formulations (mean ± SD, *n* = 2).

The physical appearance of various curcumin formulations was compared to evaluate aqueous dispersion (Fig. 1B). Free curcumin in PBS appeared as a transparent supernatant following centrifugation, consistent with its negligible aqueous solubility. Curcumin at 1 mg/mL in ethanol and DMSO formed clear yellow and orange solutions, respectively. In contrast, the 1 mg/mL Cur@LNP formulation in PBS presented as a homogeneous, opaque orange-red suspension without visible precipitation or phase separation.

Nanoscale morphology was evaluated by cryo-TEM. Cur@LNP(SM-102) formed well-dispersed spherical nanoparticles with diameters mainly below 100 nm (Fig. 1C). Cur@LNP(MC-3) and Cur@LNP(ALC-0315) showed spherical particles of comparable overall dimensions (Fig. S1A,B), indicating that all three ionizable lipid formulations generated comparable nanoscale structures.

Dynamic light scattering (DLS) measurements further confirmed the uniform particle size distributions of the three formulations. Cur@LNP(SM-102), Cur@LNP(MC-3), and Cur@LNP(ALC-0315) exhibited hydrodynamic diameters of 91, 90, and 90 nm, respectively, with narrow unimodal distributions and PDI values close to 0.1 (Fig. 1D, Fig. S1C,D). Their zeta potentials in PBS were also comparable, ranging from 1.2 to 2.1 mV (Fig. 1E), indicating that variation in ionizable lipid structure had minimal impact on the overall physicochemical properties of the Cur@LNP.

The concentration of encapsulated curcumin was determined using ultra-performance liquid chromatography (UPLC). Chromatographic analysis identified a distinct analyte peak at a retention time of approximately 0.26 min, which was absent in the empty LNP controls (Fig. 1F). A linear calibration curve was established across a concentration range of 0 to 0.25 mg/mL (Fig. S1E, S1F). Quantitative analysis showed that the different lipid formulations had similar encapsulation efficiencies of 36%, 32%, and 34% for Cur@LNP(SM-102), Cur@LNP(MC-3), and Cur@LNP(ALC-0315), respectively (Fig. 1G).

### 2.2 Antibacterial activity against planktonic *S. aureus*

**Fig. 2:**
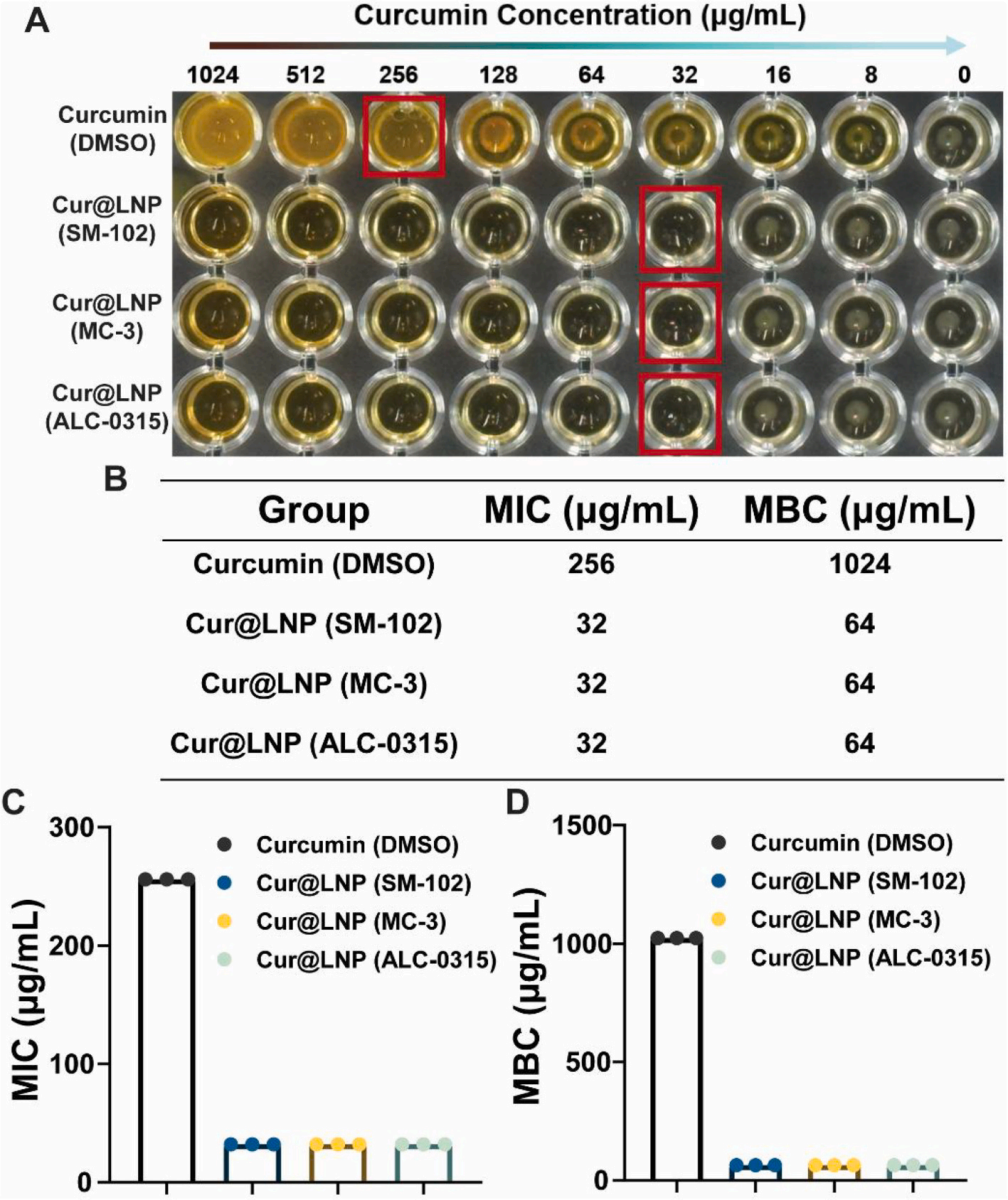
Antibacterial activity against planktonic *S. aureus*. (A) Representative broth microdilution plates showing growth inhibition by free curcumin and Cur@LNP formulations over the tested concentration range. (B) Summary table of minimum inhibitory concentration (MIC) and minimum bactericidal concentration (MBC) values. (C) MIC values for free curcumin and the three Cur@LNP formulations. (D) MBC values for free curcumin and the three Cur@LNP formulations. (mean ± SD, n = 3).

The antibacterial activity of free curcumin and Cur@LNP formulations against planktonic *S. aureus* was determined by broth microdilution (Fig. 2A). Free curcumin exhibited a minimum inhibitory concentration (MIC) of 256 μg/mL and a minimum bactericidal concentration (MBC) of 1024 μg/mL. In contrast, Cur@LNP(SM-102), Cur@LNP(MC-3), and Cur@LNP(ALC-0315) all showed an MIC of 32 μg/mL and an MBC of 64 μg/mL (Fig. 2B–D). Compared with free curcumin in DMSO, LNP encapsulation therefore corresponded to an 8-fold reduction in MIC and a 16-fold reduction in MBC. The three Cur@LNP formulations displayed identical MIC and MBC values, indicating that the enhanced activity was associated with nanoscale aqueous solubilization of curcumin rather than with differences in ionizable lipid structure.

### 2.3 Effects of Cur@LNP on biofilm formation and mature biofilm disruption

**Fig. 3:**
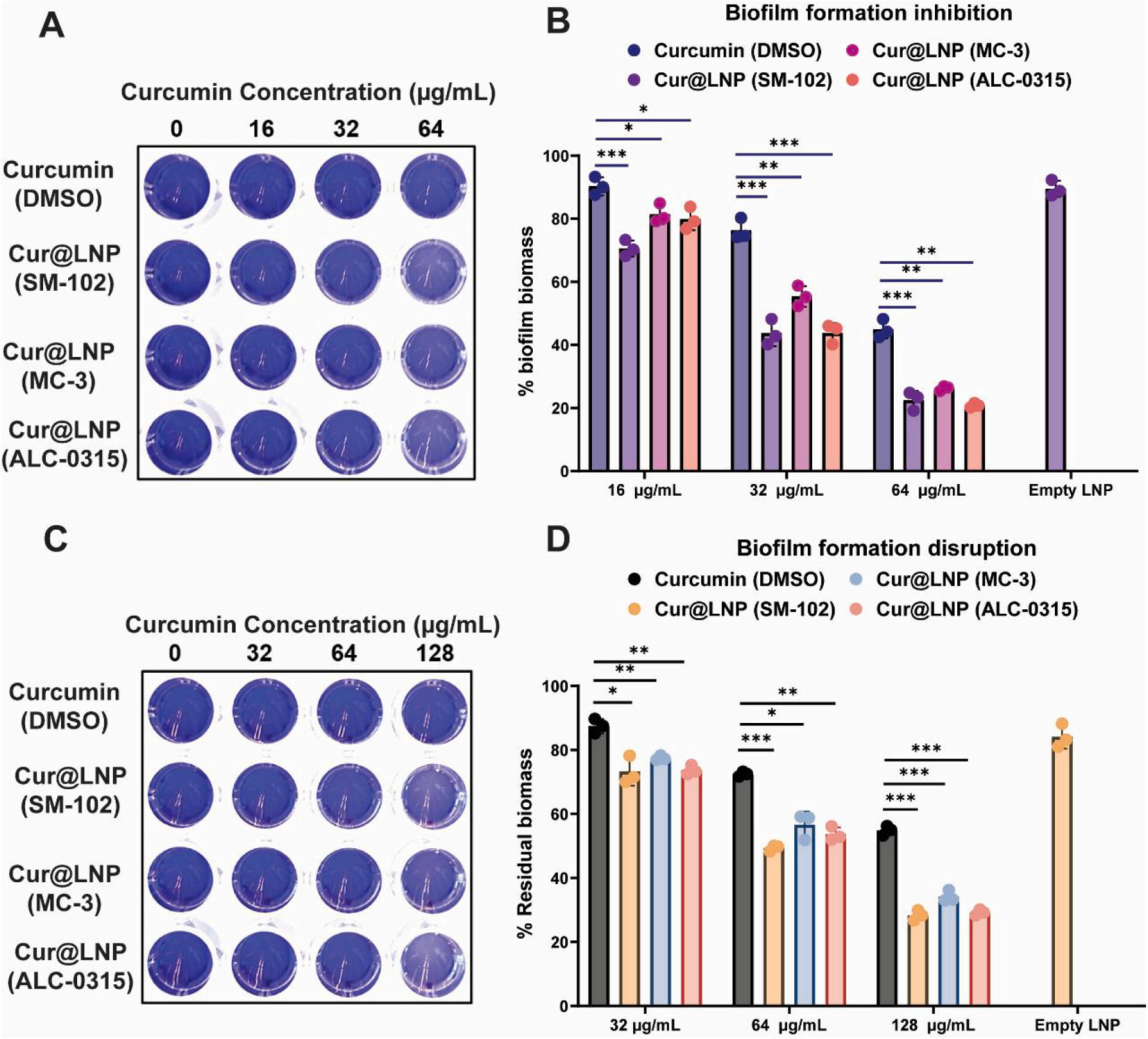
Effects of Cur@LNP on biofilm formation and disruption. (A, B) Inhibition of *S. aureus* biofilm formation assessed by crystal violet staining after 24 h co-incubation with serial concentrations of free curcumin, Cur@LNP, or empty LNP (16–64 μg/mL). (C, D) Disruption of pre-formed 24 h mature biofilms after 24 h treatment with serial concentrations of free curcumin, Cur@LNP, or empty LNP (32–128 μg/mL). (mean ± SD, *n* = 3). * *p* < 0.05, ** *p* < 0.01, *** *p* < 0.001. (For interpretation of the references to colour in this figure legend, the reader is referred to the web version of this article.)

We next examined whether the enhanced antibacterial activity of Cur@LNP extended to both the inhibition of biofilm formation and the disruption of established mature biofilms. Crystal violet staining showed that all three Cur@LNP formulations inhibited *S. aureus* biofilm formation in a concentration-dependent manner over the tested range of 16–64 μg/mL (Fig. 3A,B). Their inhibitory effects were broadly comparable across the three ionizable lipid formulations. At 64 μg/mL, Cur@LNP(SM-102), Cur@LNP(MC-3), and Cur@LNP(ALC-0315) reduced the residual biofilm biomass to approximately 20–30%, whereas free curcumin at the same concentration reduced biomass only to about 45%. In contrast, the empty LNP group maintained biomass levels above 90%, indicating that the carrier alone contributed minimally to the inhibition of biofilm formation.

We then assessed the ability of these formulations to disrupt pre-formed mature biofilms. Again, all Cur@LNP variants showed dose-dependent reductions in residual biofilm biomass across the concentration range of 32–128 μg/mL (Fig. 3C,D). At 128 μg/mL, the three Cur@LNP formulations reduced the remaining biomass to approximately 30%, while free curcumin under the same conditions left approximately 55% residual biomass. The empty LNP group showed only limited disruption, with more than 80% of the biofilm biomass remaining. Together, these results demonstrate that LNP encapsulation substantially improves the antibiofilm performance of curcumin in both preventing early biofilm establishment and promoting the disruption of established mature biofilms.

### 2.4 Viability analysis of biofilm-resident *S. aureus*

**Fig. 4:**
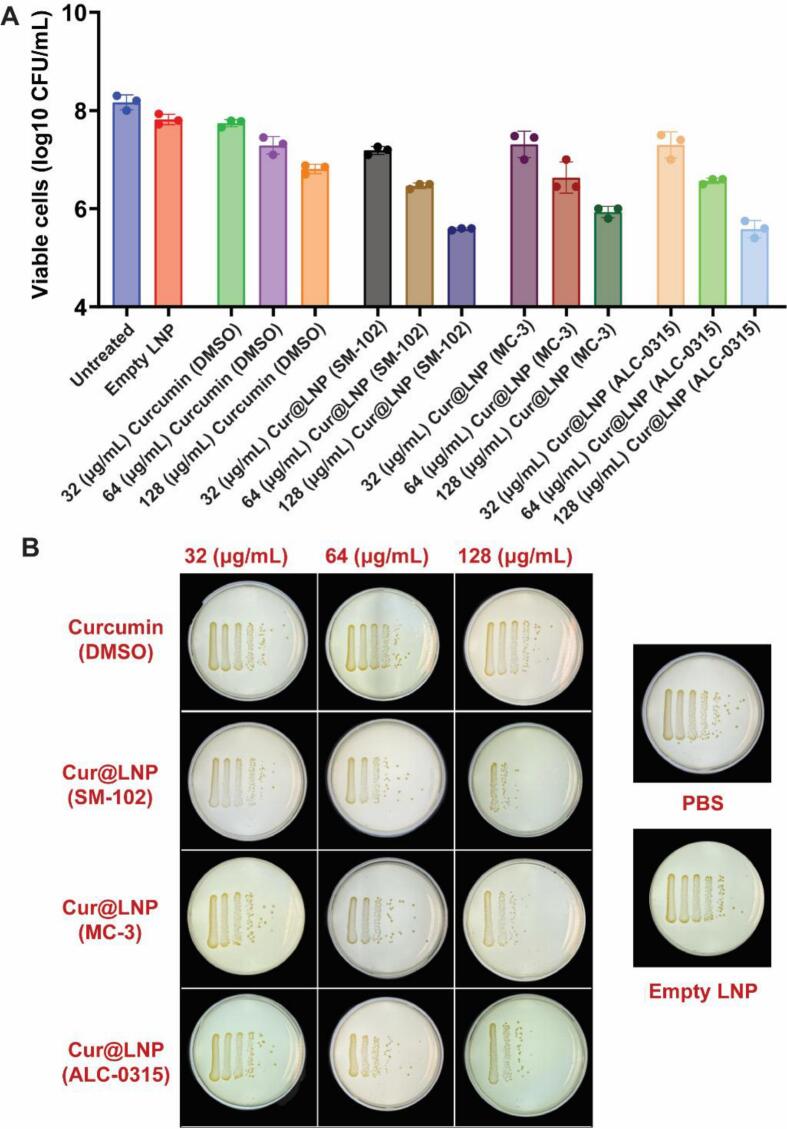
Viability of biofilm-resident *S. aureus* cells after treatment with Cur@LNP. (A) Quantitative colony-forming unit (CFU) counts (log₁₀ CFU/mL) recovered from 24 h mature biofilms following 24 h treatment with free curcumin (in DMSO), empty LNP, or the three Cur@LNP formulations at curcumin concentrations of 32, 64, and 128 μg/mL. (B) Representative agar spot plates showing colony density under the same treatment conditions. PBS and empty LNP controls are shown on the right. (mean ± SD, *n* = 3).

To further determine whether the reduction in biofilm biomass was accompanied by decreased bacterial survival, we quantified the viability of biofilm-resident *S. aureus* cells using a colony-forming unit (CFU) assay (Fig. 4A). All three Cur@LNP formulations, including Cur@LNP(SM-102), Cur@LNP(MC-3), and Cur@LNP(ALC-0315), produced a clear concentration-dependent reduction in viable cell counts. Untreated biofilms and those treated with empty LNP remained highly viable, with bacterial burdens of approximately 8 log₁₀ CFU/mL. Free curcumin showed only limited bactericidal activity, reducing viability to approximately 7.0 log₁₀ CFU/mL even at the highest tested concentration of 128 μg/mL. In contrast, treatment with Cur@LNP reduced viable counts to approximately 5.5–6.0 log₁₀ CFU/mL at the same concentration, indicating substantially greater killing of biofilm-resident cells than free curcumin. The three Cur@LNP formulations displayed broadly similar activity profiles, further indicating that the improved antibiofilm effect reflected nanoscale aqueous solubilization of curcumin rather than differences in ionizable lipid structure. These quantitative results were consistent with the agar plate spot assay, which showed a marked reduction in colony density in all Cur@LNP-treated groups compared with free curcumin at matched concentrations (Fig. 4B).

### 2.5 SEM analysis of biofilm morphology

**Fig. 5:**
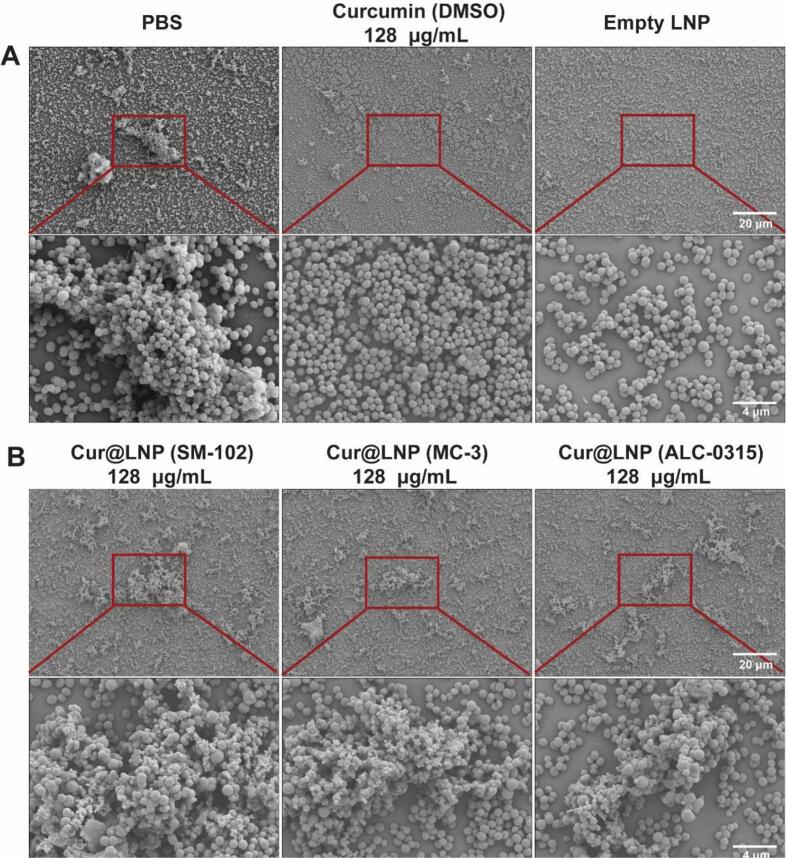
SEM analysis of biofilm morphology after treatment with Cur@LNP. (A) Representative low-magnification (1000×) and high-magnification (5000×) SEM images of untreated biofilms (PBS), free curcumin-treated biofilms (128 μg/mL in DMSO), and empty LNP-treated biofilms. (B) Representative low-magnification (1000×) and high-magnification (5000×) SEM images of biofilms treated with Cur@LNP(SM-102), Cur@LNP(MC-3), and Cur@LNP(ALC-0315) at 128 μg/mL. Red boxes indicate the regions shown at higher magnification. Scale bars: 20 μm (low magnification) and 4 μm (high magnification). (For interpretation of the references to colour in this figure legend, the reader is referred to the web version of this article.)

Scanning electron microscopy (SEM) was used to examine treatment-induced changes in biofilm architecture. Untreated biofilms (PBS control) displayed dense bacterial clusters embedded within a continuous EPS-like matrix, forming a compact and well-organized surface structure (Fig. 5A). Biofilms treated with free curcumin (128 μg/mL) still showed substantial bacterial accumulation and a relatively flat, uniform morphology, indicating only limited structural disruption under these conditions. In the empty LNP group, bacteria appeared more evenly distributed across the surface, and the continuous EPS-like coverage was less obvious than in the PBS control. We note explicitly, however, that this SEM appearance is not concordant with the crystal violet biomass and CFU data, in which empty LNP showed only minimal antibiofilm activity. The most likely explanation is the sampling bias inherent to SEM, in which only a limited number of fields are imaged after extensive dehydration and coating; we therefore caution against interpreting the empty-LNP SEM appearance as evidence of intrinsic carrier antibiofilm activity.

More pronounced morphological alterations were observed following treatment with Cur@LNP(SM-102), Cur@LNP(MC-3), and Cur@LNP(ALC-0315). At 32 μg/mL, early disruption of the biofilm matrix and the emergence of irregular bacterial aggregates were already evident (Fig. S2). At 128 μg/mL, all three Cur@LNP formulations induced marked restructuring of the biofilm, characterized by large uneven aggregates and loss of the dense, continuous surface organization seen in the control groups (Fig. 5B). High-magnification images further revealed a marked reduction in visible EPS-like material together with abundant small irregular particulates associated with the bacterial clusters, consistent with cell-associated debris. Overall, these observations indicate that Cur@LNP treatment not only reduced biofilm biomass and viability, but also substantially remodeled the architecture of mature *S. aureus* biofilms.

### 2.6 Fluorescence imaging of biofilm viability

**Fig. 6:**
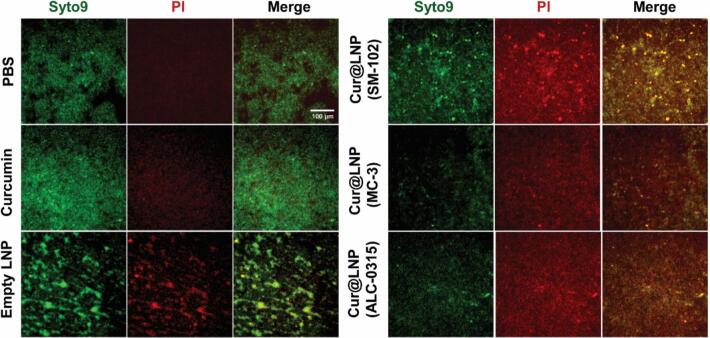
Fluorescence imaging of biofilm viability after treatment with Cur@LNP. Representative fluorescence micrographs of untreated biofilms and biofilms treated with free curcumin, empty LNP, or Cur@LNP(SM-102), Cur@LNP(MC-3), and Cur@LNP(ALC-0315). SYTO9 (green) labels total/live-associated cells, whereas propidium iodide (PI, red) marks membrane-compromised cells. Merged images are shown in the right column. Scale bar: 100 μm. (For interpretation of the references to colour in this figure legend, the reader is referred to the web version of this article.)

Live/dead fluorescence imaging further revealed clear differences in biofilm viability across the treatment groups (Fig. 6). Untreated biofilms (PBS group), as well as those treated with free curcumin or empty LNP, were dominated by strong SYTO9-positive green fluorescence with only weak PI staining, indicating that most cells remained viable and that membrane-compromised cells were limited under these conditions. In contrast, biofilms treated with Cur@LNP(SM-102), Cur@LNP(MC-3), and Cur@LNP(ALC-0315) showed markedly increased PI-positive red fluorescence together with reduced green signal throughout the biofilm structure. The three Cur@LNP formulations displayed broadly similar staining patterns, suggesting comparable effects on biofilm-resident cells regardless of ionizable lipid identity. An important caveat applies to this assay: curcumin emits in the green channel and overlaps spectrally with SYTO9 (Ex 488/Em 535), so residual curcumin associated with Cur@LNP-treated cells can contribute non-specific green signal. We therefore regard the PI channel, whose emission does not overlap with curcumin, as the more reliable viability readout here; the increased PI signal in Cur@LNP-treated biofilms is concordant with the CFU data. These visual observations are consistent with the quantitative results obtained from the CFU and biomass assays.

### 2.7 Intracellular ROS response in treated bacteria

**Fig. 7:**
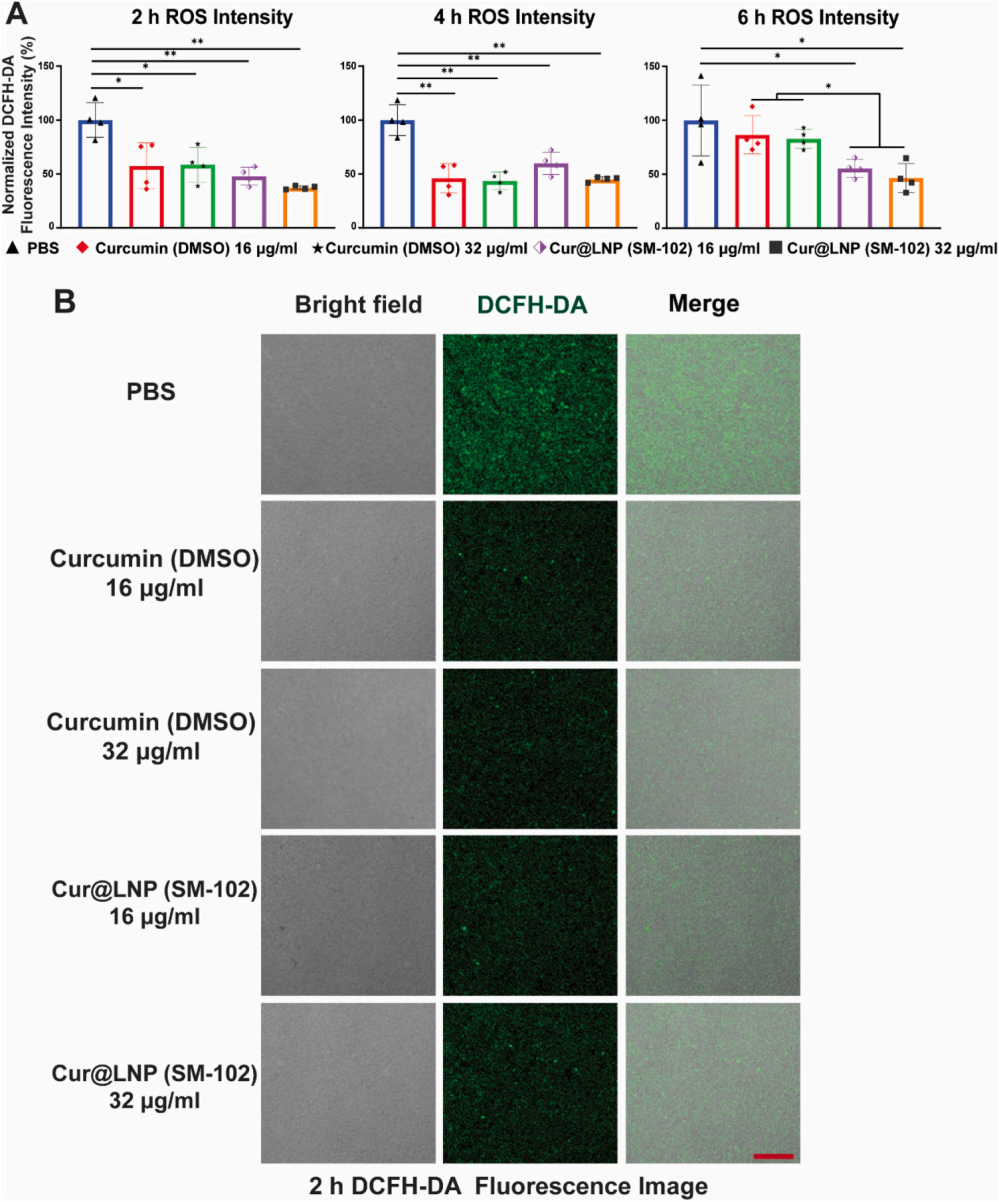
Intracellular ROS response in bacteria after treatment with free curcumin or Cur@LNP(SM-102). (A) Time-course of DCFH-DA fluorescence intensity in *S. aureus* cells exposed to free curcumin or Cur@LNP(SM-102) at the indicated concentrations, measured at 2, 4, and 6 h. PBS-treated bacteria served as the baseline control. Data are presented as mean ± SD (*n* = 3); *P < 0.05, **P < 0.01, ***P < 0.001 versus PBS control. (B) Representative fluorescence micrographs at 2 h, showing diminished DCF-associated signal in treated cells relative to the PBS control, consistent with the quantitative data in (A). Scale bar: 10 μm.

Many nanomaterial-based antimicrobial systems exert their bactericidal effects through the generation of intracellular reactive oxygen species (ROS) (Makabenta et al., 2021; Yin et al., 2026). To examine whether Cur@LNP operates through a similar pathway, intracellular ROS levels in *S. aureus* were monitored using the DCFH-DA probe (Fig. 7A). Contrary to the pattern typical of ROS-generating nanomaterials, both free curcumin and Cur@LNP(SM-102) produced lower DCF fluorescence intensities than the PBS control across all examined time points (2, 4, and 6 h). At 2 and 4 h, treated groups showed a pronounced reduction in DCF signal relative to the control, without a clear dose-dependent difference between the low- and high-dose conditions. By 6 h, the DCF signal in the free curcumin group had partially recovered toward the control level, whereas Cur@LNP(SM-102) maintained a lower signal. Fluorescence imaging was consistent with this trend, showing weaker DCF signal in both treated groups than in the PBS control (Fig. 7B, Figs. S3 and S4). We interpret these observations cautiously, because curcumin emits within the DCF detection window (Ex 495/Em 520) and is itself a well-documented antioxidant; the reduced DCF signal is therefore consistent with at least three non-exclusive explanations: a genuine reduction in intracellular oxidant burden, inner-filter or quenching artefacts from co-localized curcumin, and reduced probe retention in dying cells, which the present data cannot distinguish. We therefore restrict our conclusion to the operationally useful statement that there is no evidence of ROS overproduction in Cur@LNP-treated cells and that the bactericidal mechanism is most likely ROS-independent. Definitive characterization of the killing pathway will require orthogonal oxidant probes and curcumin-free reference standards for inner-filter correction, which are beyond the scope of the present study.

## 3 Discussion

The present study demonstrates that the encapsulation of curcumin into ionizable lipid nanoparticles (Cur@LNP) via microfluidic assembly addresses the intrinsic biopharmaceutical limitations that have long restricted its clinical utility. By mitigating the extreme hydrophobicity of curcumin and improving its presentation to *S. aureus* biofilms, this platform enhances the compound's antibiofilm activity. While free curcumin exhibits negligible aqueous solubility (<0.125 mg/L) and poor dispersibility, the microfluidic-engineered Cur@LNP provide a stable, uniform nanoscale suspension (∼ 90 nm) that ensures high bioavailability within the treatment environment. Notably, the preservation of curcumin's chemical integrity and the consistent performance observed across three structurally distinct ionizable lipids point to a cargo-dominant mode of action, in which the lipid carrier serves chiefly to render curcumin aqueous-deployable. This structural and functional reproducibility contrasts favorably with conventional solid lipid nanoparticle (SLN) systems (Sandhu et al., 2021), which are often plagued by broad size polydispersity (15 to >500 nm) and batch-to-batch variability. Consequently, the marked reductions in MIC (8-fold lower) and MBC (16-fold lower), alongside the superior disruption of mature biofilms, are most likely explained by efficient aqueous solubilization of curcumin and the resulting high local availability of the solubilized drug, rather than by any intrinsic engagement of the lipid carrier with the biofilm matrix.

Beyond passive solubilization, it has been hypothesized that ionizable lipids might engage the mildly acidic microenvironment of *S. aureus* biofilms (pH 5.5–6.5), where their headgroups can become protonated and strengthen electrostatic interactions with the negatively charged EPS matrix, such as teichoic acids and extracellular DNA (Deiss-Yehiely et al., 2023; Kwiecinski et al., 2019; Zhou and Fey, 2020). However, the present study was not designed to test this, and our data neither support nor exclude such a mechanism: the decisive experiment, a matched LNP in which the ionizable lipid is replaced by a permanently neutral helper lipid carrying the same curcumin payload, was not performed here. We therefore treat any ionizable-lipid-specific contribution as an untested hypothesis rather than an established mechanism, and base our conclusions on the cargo-dominant behavior that the data do support.

The empty-LNP condition illustrates how difficult it is to infer mechanism from morphology alone: although empty LNPs produced some apparent reduction in visible EPS coverage by SEM, this was not accompanied by meaningful reductions in biomass or viable cell counts. As noted in the Results, we attribute the empty-LNP SEM appearance to sampling and preparation artefacts rather than to intrinsic carrier activity, and we do not interpret it as evidence that the ionizable lipids actively destabilize the EPS matrix. Consistent with this cargo-dominant view, the antibiofilm effect sizes we observe are broadly comparable to those reported for other curcumin nanocarriers, including liposomal, solid lipid, chitosan, and mesoporous silica systems (Bhatia et al., 2021; Ma et al., 2020; Memar et al., 2025; Sandhu et al., 2021; Wu et al., 2022), which likewise act mainly by improving the aqueous presentation of curcumin. Framed against these systems, the advantage of the present platform is pharmaceutical rather than mechanistic: microfluidic assembly with clinically established ionizable lipids produced highly uniform particles (PDI ∼0.1) in a single, reproducible, and readily scalable step and yielded an aqueous, organic-solvent-free suspension after dialysis, in contrast to the broader size dispersity and batch-to-batch variability often reported for solid lipid and some liposomal curcumin preparations (Sandhu et al., 2021). Its limitations should, however, be stated plainly: the encapsulation efficiency achieved here (32–36%) is moderate rather than high; the antibiofilm effect sizes were comparable to, not greater than, those of existing curcumin nanocarriers (Medaglia et al., 2024; Salama et al., 2024); and, because all three ionizable lipids behaved identically, we found no evidence that the ionizable chemistry contributes beyond solubilization in this system. The simultaneous reduction in total biomass and viable cell burden in our data is consistent with curcumin's known multi-target activity once it is delivered in a stable, aqueous-deployable form, even in a heterogeneous environment where restricted diffusion and tolerant subpopulations markedly reduce antimicrobial susceptibility.

Notably, we found no evidence that the enhanced antibacterial activity of Cur@LNP is driven by intracellular ROS generation. Although ROS overproduction is often proposed as a killing mechanism in nanomaterial-based systems, DCFH-DA signals can be influenced by multiple forms of cellular stress and, as discussed above, by curcumin's own fluorescence and antioxidant activity, and should not be interpreted as definitive evidence of a single pathway (Makabenta et al., 2021; Yin et al., 2026). Our data therefore do not support ROS overproduction as the dominant mechanism underlying Cur@LNP activity. Instead, the observed effects are more consistent with ROS-independent, multifactorial processes. Previous studies have shown that curcumin can disrupt bacterial membrane integrity and permeability (Tyagi et al., 2015), inhibit assembly of the essential cell division protein FtsZ (Rai et al., 2008), and downregulate quorum-sensing networks involved in virulence and biofilm development (Teow et al., 2016). By improving curcumin delivery and likely increasing its local concentration within the restricted biofilm environment, LNP encapsulation may enable these established multi-target mechanisms to operate more effectively.

A noteworthy comparison can be drawn with established mRNA@LNP systems. In mRNA delivery, even subtle variations in lipid pKa or formulation composition are known to significantly influence endosomal escape and transfection efficiency (Simonsen and Larsson, 2025; Zheng et al., 2023), making lipid properties a major determinant of therapeutic outcome. In contrast, the present antibacterial application yielded nearly identical in vitro efficacy across all tested endpoints despite the use of three structurally distinct ionizable lipids. This contrast suggests that, once the LNP platform overcomes curcumin's solubility and permeability limitations, the intrinsic bioactivity of the cargo itself becomes the primary driver of antibiofilm activity in this in vitro system. Practically, this means the platform can accommodate different clinical-grade ionizable lipids without loss of activity, which simplifies formulation. Whether this cargo-dominant behavior is maintained in vivo, and against other pathogens, remains to be investigated.

Although these in vitro results highlight the therapeutic potential of Cur@LNP, several specific limitations must be addressed before clinical translation. First, the biological evaluation was deliberately confined to a single, well-characterized, methicillin-sensitive laboratory strain (*S. aureus* USA300 LAC). This is appropriate for a controlled proof-of-concept comparison of lipid chemistries, but it limits the translational reach of our conclusions. Clinical biofilm-associated infections are frequently caused by methicillin-resistant *S. aureus* (MRSA) and by diverse clinical isolates whose matrix composition, antibiotic tolerance, and architecture differ from those of a single laboratory strain, and many device- and wound-associated infections are Gram-negative or polymicrobial, settings in which outer-membrane permeability barriers and interspecies interactions can substantially alter both nanoparticle penetration and curcumin susceptibility (Tong et al., 2015). Because we did not test MRSA isolates, clinical strains, Gram-negative pathogens, or mixed-species biofilms, the generality of the aqueous-solubilization benefit reported here remains to be established, and our findings should be read as a mechanism-focused proof of concept rather than as evidence of broad antibiofilm efficacy. In addition, in vitro biofilm models cannot reproduce the physiological and immunological complexity of a living host, in which further biological barriers govern nanoparticle transport, retention, and clearance (Blanco et al., 2015). Second, protein corona formation remains an important variable: upon exposure to physiological fluids (e.g., wound exudate), nanoparticles rapidly adsorb biomolecules that can alter their physicochemical identity and targeting behavior (Wang et al., 2023; Xiao et al., 2022), potentially reducing the ability of LNP to penetrate deep-seated biofilms in vivo. Third, long-term storage stability and release kinetics under physiological shear and pH conditions were not assessed here. Mammalian cell compatibility and cytotoxicity were likewise not evaluated; we note, however, that in vitro cytotoxicity at the concentrations required for antibiofilm activity is unlikely to predict the in vivo therapeutic window, because local delivery, pharmacokinetics, exposure time, and clearance differ substantially from static in vitro exposure. The therapeutic index of Cur@LNP is therefore best established in vivo. Deeper molecular insight into curcumin's intracellular targets and its modulation of quorum-sensing pathways would also benefit from transcriptomic or proteomic analyses. Most importantly for the central question of this study, we did not include a matched non-ionizable lipid control; without it, the relative contributions of aqueous solubilization and of any ionizable-lipid-specific effect cannot be separated. Addressing these gaps (in vivo pharmacokinetics, tissue penetration, biocompatibility, and the matched-control experiment outlined above) will be necessary to define the full therapeutic scope of the platform; we therefore regard the present study as a foundation for that work rather than a definitive characterization.

In summary, microfluidic-assembled ionizable lipid nanoparticles provide a reproducible, scalable platform that enhances the antibiofilm utility of curcumin chiefly by improving its aqueous solubilization. Across three structurally distinct, clinically established ionizable lipids, the antibiofilm activity was dominated by the cargo rather than by lipid identity, identifying these LNPs as an effective solubilization platform for poorly soluble natural antimicrobials. Whether the ionizable lipid contributes beyond solubilization, through the proposed pH-responsive engagement of the biofilm matrix, remains to be established, and defines the central question for future mechanistic and in vivo studies.

## 4 Conclusion

In summary, our findings show that microfluidic-assembled ionizable lipid nanoparticles provide an effective platform for overcoming the formulation and biofilm-related barriers that limit the antibacterial utility of curcumin. Cur@LNP displayed improved aqueous solubilization and consistently outperformed free curcumin across planktonic antibacterial assays and multiple biofilm endpoints, including biofilm inhibition, mature biofilm disruption, viable-cell reduction, and structural remodeling. The indistinguishable outcomes across three structurally distinct, clinically established ionizable lipids indicate that, in this in vitro system, antibiofilm activity is driven chiefly by the cargo rather than by lipid identity, although whether the lipid contributes beyond solubilization remains to be tested directly. Overall, these findings position ionizable LNPs as a reproducible and scalable delivery system for poorly soluble natural antimicrobials, and a practical starting point for the in vivo and mechanistic studies needed to establish their therapeutic value in biofilm-associated infections.

## 5 Materials and methods

### 5.1 Materials

Curcumin (≥94% purity), the ionizable lipids SM-102, MC-3, and ALC-0315, 1,2-distearoyl-sn-glycero-3-phosphocholine (DSPC), cholesterol, and 1,2-dimyristoyl-rac-glycero-3-methoxypolyethylene glycol-2000 (DMG-PEG2000) were purchased from commercial suppliers (Sigma-Aldrich or Avanti Polar Lipids). Ultrapure water was used throughout. Curcumin-loaded ionizable lipid nanoparticles are abbreviated as Cur@LNP; when a specific ionizable lipid is used, the formulation is denoted as Cur@LNP(SM-102), Cur@LNP(MC-3), or Cur@LNP(ALC-0315).

### 5.2 Bacterial strain and growth conditions

*Staphylococcus aureus* USA300 LAC (AH1263) was used for all experiments (Boles et al., 2010). The strain was streaked onto tryptic soy agar (TSA) plates and incubated overnight at 37 °C. A single colony was inoculated into tryptic soy broth (TSB) and cultured overnight at 37 °C with shaking (250 rpm). Log-phase cultures were obtained by diluting the overnight culture 1:100 in fresh TSB and growing for 3 h at 37 °C.

### 5.3 Preparation of Cur@LNP

Cur@LNP formulations were prepared by microfluidic mixing using a NanoAssemblr Benchtop system (Precision NanoSystems). The lipid mixture consisted of the respective ionizable lipid (SM-102, MC-3, or ALC-0315), DSPC, cholesterol, and DMG-PEG2000 in a molar ratio of 50:10:38.5:1.5. Curcumin and lipids were co-dissolved in ethanol at a total lipid concentration of 10 mM (curcumin concentration adjusted to achieve a final encapsulated concentration of 1 mg/mL in the formulated suspension). The aqueous phase was 25 mM sodium acetate buffer (pH 4.0). The ethanol and aqueous phases were mixed at a flow-rate ratio of 1:3 (total flow rate 9 mL/min) (Fig. 1A). The resulting suspensions were immediately dialyzed against PBS (pH 7.4) using a 10 kDa MWCO cassette (Thermo Fisher Scientific, USA) for 16 h at 4 °C with three buffer exchanges to remove residual ethanol and unencapsulated curcumin. Empty LNP (no curcumin) were prepared identically. The purified Cur@LNP suspension was concentrated to the desired curcumin concentration via centrifugal ultrafiltration using a 100 kDa molecular weight cut-off (MWCO) membrane. Lipid content of Cur@LNP and empty LNP was determined by quantifying cholesterol using a Cholesterol/Cholesteryl Ester Quantitation Assay Kit (ab65359, Abcam) according to the manufacturer's instructions, to confirm comparable lipid concentrations between formulations. Formulations were stored at 4 °C.

### 5.4 Physicochemical characterization

Hydrodynamic diameter and polydispersity index (PDI) were measured by dynamic light scattering (DLS), and zeta potential was measured by electrophoretic light scattering using a Zetasizer Nano ZS (Malvern Panalytical). Samples were diluted 1:20 in PBS and analyzed at 25 °C in triplicate. Morphology was assessed by cryo transmission electron microscopy (cryo-TEM). Samples were vitrified using a Vitrobot Mark IV and imaged under liquid nitrogen on the same instrument at 200 kV. Curcumin concentration and encapsulation efficiency (EE) were determined by ultra-performance liquid chromatography (UPLC) on a Waters Acquity system equipped with a BEH C18 column (2.1 × 50 mm, 1.7 μm, Waters, USA). The mobile phase was Ethanol/Water (4/1), flow rate 0.5 mL/min, detection at 425 nm. Curcumin eluted at approximately 0.26 min. Because curcumin elutes close to the column void volume under these conditions, this UPLC assay is best regarded as a rapid quantitative dissolution measurement rather than a chromatographic separation; method specificity was confirmed by the absence of a co-eluting peak in empty-LNP controls (Fig. 1F). A linear calibration curve (0–0.25 mg/mL, R^2^ > 0.99) was established. To quantify the encapsulated curcumin, an aliquot of the purified Cur@LNP suspension (obtained after dialysis and centrifugal ultrafiltration, which together remove ethanol and any non-encapsulated or aggregated curcumin) was diluted in the ethanol/water (4/1) mobile phase to fully dissolve the lipid particles and release the entrapped curcumin, which was then quantified against the calibration curve described above. The encapsulation efficiency (EE) was calculated as the amount of curcumin recovered in the purified nanoparticles relative to the total amount of curcumin initially added to the ethanol (lipid) phase during formulation, using the following formula:

$$\mathrm{EE}\;\left(\%\right)=\frac{\left[\text{Encapsulated curcumin}\right]}{\left[\text{Total curcumin}\right]}\times 100\%.$$

### 5.5 Minimum inhibitory concentration (MIC) and minimum bactericidal concentration (MBC)

MIC and MBC were determined by the broth microdilution method according to CLSI guidelines (Balouiri et al., 2016). Twofold serial dilutions of free curcumin or Cur@LNP, corresponding to final curcumin concentrations of 4–1024 μg/mL, were prepared in 96-well plates containing cation-adjusted Mueller–Hinton broth. Log-phase *S. aureus* cultures were added to achieve a final inoculum of approximately 5 × 10^5 CFU/mL. Plates were incubated statically at 37 °C for 18–24 h. The MIC was defined as the lowest concentration showing no visible bacterial growth. For MBC determination, 10 μL aliquots from wells without visible growth were spotted onto tryptic soy agar plates and incubated at 37 °C for 24 h. The MBC was defined as the lowest concentration resulting in ≥99.9% bacterial killing, as indicated by the absence of colony growth.

### 5.6 Biofilm formation inhibition assay

Overnight bacterial cultures were diluted to approximately 1 × 10^5 CFU/mL in TSB supplemented with 0.5% (*w*/*v*) glucose. Aliquots (200 μL) were added to 96-well plates together with serial dilutions of free curcumin, Cur@LNP, or empty LNP to give final curcumin concentrations of 16–64 μg/mL. Plates were incubated statically at 37 °C for 24 h to allow biofilm formation. The wells were then gently washed twice with PBS to remove non-adherent cells, heat-fixed at 60 °C for 60 min, and stained with 0.1% (w/v) crystal violet for 15 min. After rinsing and air-drying, the bound dye was solubilized in 33% (*v*/v) acetic acid and quantified by measuring absorbance at 595 nm.

### 5.7 Mature biofilm disruption assay

Biofilms were first established for 24 h under the same conditions described above, without the addition of test compounds. After washing twice with PBS, the pre-formed biofilms were treated with fresh medium containing free curcumin, Cur@LNP, or empty LNP at final curcumin concentrations of 32–128 μg/mL, followed by incubation at 37 °C for an additional 24 h. Residual biofilm biomass was then quantified by crystal violet staining as described above.

### 5.8 Biofilm viability (CFU assay)

Following the mature biofilm disruption treatment, the biofilms were washed twice with PBS and dispersed by sonication (40 kHz, 5 min, on ice), followed by vigorous pipetting. Serial 10-fold dilutions of the resulting suspensions were plated onto TSA plates and incubated at 37 °C for 18–24 h for colony-forming unit (CFU) enumeration.

### 5.9 Scanning electron microscopy (SEM)

Biofilms were established on poly-l-lysine-coated 12 mm glass coverslips and treated as indicated. Samples were fixed for 24 h at room temperature in 2% formaldehyde, 0.5% glutaraldehyde, and 0.15% ruthenium red prepared in 0.1 M phosphate buffer (pH 7.4), then washed twice with the same buffer. After graded ethanol dehydration (50%, 70%, 80%, 95%, and 100% twice), the samples were transferred into HMDS (50% HMDS in ethanol, followed by 100% HMDS twice) and left to air-dry overnight. The dried specimens were sputter-coated with an 8 nm gold layer using a Quorum Q150R S and imaged on a Zeiss Gemini 450 SEM at 6–7 kV with a working distance of 15 mm.

### 5.10 Fluorescence imaging of biofilm viability

Biofilms formed on glass coverslips were treated as described above, washed with PBS, and stained using the LIVE/DEAD BacLight bacterial viability kit with SYTO9 (6 μM) and propidium iodide (PI, 30 μM) (Thermo Fisher Scientific, Waltham, MA, USA) for 15 min in the dark. The samples were then rinsed gently and imaged by fluorescence microscopy (Leica Thunder imaging system). For fluorescence imaging, excitation/emission settings of 488/535 nm were used for SYTO9 and 535/617 nm for PI.

### 5.11 Intracellular ROS measurement

Intracellular reactive oxygen species (ROS) levels were quantified using the 2′,7′-dichlorodihydrofluorescein diacetate (DCFH-DA) probe (Sigma-Aldrich, St. Louis, MO, USA). Briefly, bacteria were pre-incubated with 10 μM DCFH-DA for 30 min at 37 °C in the dark. Following incubation, the cells were treated with free curcumin or Cur@LNP(SM-102) at the indicated concentrations. Fluorescence intensity was monitored at 2, 4, and 6 h using a microplate reader at excitation/emission wavelengths of 495/520 nm, respectively. PBS-treated samples served as the negative control. For qualitative analysis, representative samples were imaged using a Leica THUNDER imaging system.

### 5.12 Statistical analysis

All experiments were performed with at least three independent biological replicates, each including technical duplicates or triplicates. Data are presented as mean ± standard deviation (SD). Statistical analyses were performed using one-way analysis of variance (ANOVA), followed by Dunnett's or Tukey's post hoc test, using GraphPad Prism 10. A value of *P* < 0.05 was considered statistically significant.

## Declaration of generative AI and AI-assisted technologies in the manuscript preparation process

During the preparation of this work the authors used Claude, Gemini, and ChatGPT in order to assist with grammar correction, rephrasing, and improving the clarity of the language. After using these tools, the authors reviewed and edited the content as needed and take full responsibility for the content of the publication.

## CRediT authorship contribution statement

**Xin Jin:** Writing – original draft, Validation, Project administration, Methodology, Investigation, Formal analysis, Data curation, Conceptualization. **Zhentao Xing:** Writing – review & editing, Validation, Methodology, Investigation, Data curation. **Geng Yang:** Writing – review & editing, Methodology, Formal analysis. **Harrie Weinans:** Writing – review & editing, Supervision, Resources, Funding acquisition. **Jaqueline Lourdes Rios:** Writing – review & editing, Supervision, Resources. **Joost P.G. Sluijter:** Writing – review & editing, Supervision, Resources. **Raymond M. Schiffelers:** Writing – review & editing, Supervision, Resources, Funding acquisition. **Zhiyong Lei:** Writing – review & editing, Supervision, Resources. **Zijian Ye:** Writing – review & editing, Validation, Methodology, Investigation, Data curation, Conceptualization.

## Funding

This publication is part of the project DARTBAC (with project number NWA.1292.19.354) of the research program NWA-ORC, which is (partly) financed by the Dutch Research Council (NWO). This work was supported by the 10.13039/501100000780European Union's Horizon 2020 research and innovation program [Grant Agreement No. 825828].The funders had no role in study design, data collection, analysis, interpretation, or the decision to submit the article for publication.

## Declaration of competing interest

The authors declare that they have no known competing financial interests or personal relationships that could have appeared to influence the work reported in this paper.

## Data Availability

Data will be made available on request.

## Appendix A Supplementary data

Supplementary data: Figures S1–S4. Additional cryo-TEM images, DLS size distributions, UPLC chromatograms and the curcumin calibration curve for Cur@LNP (Fig. S1); high-magnification SEM images of Cur@LNP-treated S. aureus biofilms (Fig. S2); and representative fluorescence micrographs of intracellular ROS at 4 h and 6 h (Figs. S3 and S4)
