# Supplementary material for: Microfluidic-assembled curcumin ionizable lipid nanoparticles enhance aqueous delivery and antibiofilm activity against Staphylococcus aureus
Source: Int J Pharm X. 2026 Aug 3;12:100630. doi: 10.1016/j.ijpx.2026.100630 (PMC13463254; doi:10.1016/j.ijpx.2026.100630)
Supplement: Supplementary file 1 [file mmc1.docx]

**Supplementary materials of**

**Microfluidic-Assembled Curcumin Ionizable Lipid Nanoparticles Enhance Aqueous Delivery and Antibiofilm Activity against *Staphylococcus aureus***

Xin Jin^1#^, Zhentao Xing^2#^, Geng Yang^3^, Harrie Weinans^4^, Jaqueline Lourdes Rios^4^, Joost PG Sluijter^3,5^, Raymond M. Schiffelers^1*^, Zhiyong Lei^1*^, Zijian Ye^4*^

**Affiliations:**

1 Laboratory of CDL Research, University Medical Center Utrecht, Utrecht 3508 GA, The Netherlands

2 Department of Urology, University Medical Center Utrecht, Utrecht 3584 CX, The Netherlands

3 Laboratory of Experimental Cardiology, Department of Heart & Lungs, University Medical Center Utrecht, Utrecht 3584 CX, The Netherlands

4 Department of Orthopaedics, University Medical Center Utrecht, Utrecht 3584 CX, The Netherlands

5 UMC Utrecht Regenerative Medicine Center, Circulatory Health Research Center, University Medical Center Utrecht, Transplantation Center Utrecht, Utrecht University, Utrecht 3508 GA, The Netherlands

\# These authors contributed equally to this work: Xin Jin, Zhentao Xing

**Corresponding authors:**

Raymond M. Schiffelers (r.schiffelers@umcutrecht.nl)

Zhiyong Lei (z.lei@umcutrecht.nl)

Zijian Ye (z.ye@umcutrecht.nl)

**Submission contact:** Xin Jin (x.jin@umcutrecht.nl), handling correspondence on behalf of the corresponding authors.

**Figure S1.** Additional cryo-TEM images (A, B), DLS size distributions (C, D), representative UPLC chromatograms of curcumin at different concentrations (E), and the corresponding calibration curve (F).

**Figure S2**. Additional high-magnification SEM images of Cur@LNP-treated biofilms. (scale bar: 4 µm)

**Figure S3**. Representative fluorescence micrographs showing reduced ROS signal in treated cells at 4 h (scale bar: 10 µm).

**Figure S4**. Representative fluorescence micrographs showing reduced ROS signal in treated cells at 6 h (scale bar: 10 µm).
